# Robust and accurate prediction of self-interacting proteins from protein sequence information by exploiting weighted sparse representation based classifier

**DOI:** 10.1186/s12859-022-04880-y

**Published:** 2022-12-01

**Authors:** Yang Li, Xue-Gang Hu, Zhu-Hong You, Li-Ping Li, Pei-Pei Li, Yan-Bin Wang, Yu-An Huang

**Affiliations:** 1 School of Computer Science and Information Engineering, Hefei University of Technology, Hefei, 230601 China; 2 School of Computer Science, Northwestern Polytechnical University, Xi’an, 710129 Shaanxi China; 3 College of Grassland and Environment Sciences, Xinjiang Agricultural University, Urumqi, 830052 China; 4 School of Cyber Science and Technology, Zhejiang University, Hangzhou, 310027 China; 5 Guangxi Academy of Sciences, Nanning, 530007 Guangxi China

**Keywords:** Self-interacting proteins, Protein sequence, Gray level co-occurrence matrix, Sparse representation

## Abstract

**Background:**

Self-interacting proteins (SIPs), two or more copies of the protein that can interact with each other expressed by one gene, play a central role in the regulation of most living cells and cellular functions. Although numerous SIPs data can be provided by using high-throughput experimental techniques, there are still several shortcomings such as in time-consuming, costly, inefficient, and inherently high in false-positive rates, for the experimental identification of SIPs even nowadays. Therefore, it is more and more significant how to develop efficient and accurate automatic approaches as a supplement of experimental methods for assisting and accelerating the study of predicting SIPs from protein sequence information.

**Results:**

In this paper, we present a novel framework, termed GLCM-WSRC (gray level co-occurrence matrix-weighted sparse representation based classification), for predicting SIPs automatically based on protein evolutionary information from protein primary sequences. More specifically, we firstly convert the protein sequence into Position Specific Scoring Matrix (PSSM) containing protein sequence evolutionary information, exploiting the Position Specific Iterated BLAST (PSI-BLAST) tool. Secondly, using an efficient feature extraction approach, i.e., GLCM, we extract abstract salient and invariant feature vectors from the PSSM, and then perform a pre-processing operation, the adaptive synthetic (ADASYN) technique, to balance the SIPs dataset to generate new feature vectors for classification. Finally, we employ an efficient and reliable WSRC model to identify SIPs according to the known information of self-interacting and non-interacting proteins.

**Conclusions:**

Extensive experimental results show that the proposed approach exhibits high prediction performance with 98.10% accuracy on the yeast dataset, and 91.51% accuracy on the human dataset, which further reveals that the proposed model could be a useful tool for large-scale self-interacting protein prediction and other bioinformatics tasks detection in the future.

## Introduction

Cells are the fundamental units of the structure and function in the organism. Typically, a small cell may contain many thousands of proteins. Protein, as an essential substance in cells, affects the cells by interacting with other components, which plays a vital role in maintaining normal physiological functions in living organisms. In these interactions, protein–protein interactions (PPIs) have always been a hot spot for studying biological processes and thus have received widespread attention from more scholars. To fully understand both cell functions and biological phenomena, it is imperative to consider such an interesting and key question, namely, whether and how proteins interact with their partners, which is a special type of PPIs that are called self-interacting proteins (SIPs). SIPs are those proteins that have more than two copies of the protein that can actually interact with each other, among which the two SIP partners are the same copies and that can be expressed as the same gene. Hence, it can cause problems in the formation of homo-oligomer [1, 2]. Researchers found that homo-oligomerization has been an essential function of many biological processes, particularly in enzyme activation, signal transduction, immune response and gene expression regulation [3–5]. Previous works have demonstrated that SIPs play a critical role in the evolution of cellular physiological functions and protein interaction networks (PINs), which will also facilitate us to enhance our understanding of cellular functions through a systematic approach as well as provide a theoretical basis for developing novel drug targets and drug design methods [6–8]. Additionally, SIPs can effectively improve the stability of proteins and avoid the denaturation of proteins through decreasing its surface area. Consequently, it has become increasingly important to design an efficient and reliable computational method as a complement to the traditional experimental method for identifying SIPs.

Previously, numerous studies have been devoted to develop computational-based approaches for PPIs prediction [9, 10]. For instance, Wang et al. [11] presented a computational method for predicting PPIs from protein sequences by combining the Zernike moments descriptor with the probabilistic classification vector machines model. Zahiri et al. [12] introduced a sequence-based evolutionary information model named PPIevo for predicting PPIs, which extracts features from the position-specific scoring matrix of protein sequences and the results show that it has a better prediction performance in detecting PPIs. Huang et al. [13] proposed a novel computational method to predict PPIs. The proposed method was applied to global encoding on substitution matrix representation of protein sequences with the combination of weighted sparse representation classifier. In order to construct a sequence-based multiple classifier system for identifying PPIs, Xia et al. [14] adopted auto-correlation descriptors as a feature extraction algorithm to code both interacting and non-interacting protein pairs. An et al. [15] reported a method that used gray wolf optimization algorithm for generating feature vectors from protein sequences and adopted K-fold cross-validation as well as the relevance vector machine classifier to identify PPIs by considering the features of local and global of protein interaction positions. Shi et al. [16] proposed an efficient computational model based on protein sequences to predict PPIs by integrating correlation coefficient feature representation of protein sequences and support vector machine (SVM) classifier. Wang et al. [17] developed a pure biological language processing approach for the prediction of PPIs, which employed biological sequence features called bio-to-vector (Bio2Vec) as a novel representation and used the convolution neural network (CNN) to execute classification. Liu et al. [18] presented an approach called SPAR (self-interacting Protein Analysis serveR) to predict PPIs. The SPAR combined critical residues substitution (CRS) and tenfold cross-validation with the random forest algorithm for detecting PPIs. The SPAR obtained a good prediction performance in cross-species application. Nevertheless, although these methods can be used for predicting PPIs and have yielded some good prediction results, they also have some limitations. First, these computational models of PPIs are not fully applicable to the prediction of SIPs, and even if they can be predicted, the prediction results are usually not very effective. Second, compared with the computational models of PPIs, the existing computational models of SIPs are relatively few, and these models tend to ignore the problem of unbalanced data sets in SIPs, so the prediction performance of the models can be further improved by introducing reasonable computer techniques to deal with the problem of unbalanced data in the task of SIPs prediction. Therefore, it is particularly important to explore efficient and stable computational methods for large-scale SIPs detection by automated means nowadays.

In this paper, we put forward a novel computational scheme that integrates the gray level co-occurrence matrix (GLCM) feature extraction algorithm, adaptive synthetic (ADASYN) technique with weighted sparse representation based classification (WSRC) model for predicting SIPs from protein primary sequence information. We first transformed the SIPs sequences into position specific scoring matrices (PSSM) that can contain protein evolutionary information. Second, a novel feature descriptor called gray level co-occurrence matrix is employed to abstract salient and invariant feature vectors from the PSSM, and then the ADASYN technique is applied to balance the training dataset to create new feature vectors for classification. Finally, the optimized features are fed into the WSRC model to detect whether a protein is interacting or non-interacting with itself. The proposed model was performed on two benchmark SIPs datasets including yeast and human, which obtained high average accuracies of 98.10% and 91.51% using the five-fold cross-validation, respectively. Meanwhile, the comparison results, validated experimentally by the SVM-based method and other existing methods, reveal that the proposed model is effective and robust, and it is suitable for detecting potential SIPs.

The rest of the paper is organized as follows. In “Methods” section, we first introduce a highly reliable dataset for SIPs prediction, then give the evolutionary matrix representation, the position specific scoring matrix, and finally describe the proposed computational methods, which include adaptive synthetic sampling approach, gray level co-occurrence matrix (GLCM) feature descriptors, and weighted sparse representation based classification. In “Results and discussion” section, we give evaluation metrics for the prediction of SIPs, and discuss the prediction performance of the proposed model as well as compare it with other existing computational models through several comparison experiments. In “Conclusion” section, we give the conclusion of the paper.

## Methods

### Dataset

In this experiment, to construct a high reliability data source, we accessed the Uniprot database and downloaded human protein sequence data with a number of 20,199 from the database [19]. As we know, protein–protein interaction data can be collected from various databases, including BioGRID [20], DIP [21], InnateDB [22], IntAct [23] and MatrixDB [24]. In order to obtain the SIPs dataset required for the experiment, it is necessary to collect the PPIs data that can interact with itself in advance, that is, the data only contain the identical two interacting protein sequences, whose interaction type is referred to as ‘direct interaction’ in relational databases. In this way, we finally established 2994 human self-interacting protein sequences that were used to construct the experimental datasets.

To evaluate the prediction performance of the proposed model scientifically and efficiently, we screened the 2994 human SIPs datasets by the following three steps: [18] Firstly, we only retained those protein sequences with a length of more than 50 residues and less than 5000 residues from the whole human proteome. Secondly, to ensure the high quality of the SIPs data, we constructed the positive dataset used for this experiment, which has to meet at least one of the following three conditions: (a) There have been reported at least two publications for protein self-interaction; (b) The protein is referred to as a homo-oligomer (containing homodimer and homotrimer) in UniProt; (c) It is revealed by at least one small-scale experiment or two types of large-scale experiments. Finally, for constructing the human negative dataset, we remove all known SIPs from the entire human proteome (including proteins annotated as more extensive ‘physical association’ and ‘direct interaction’. Hence, the human dataset in this experiment consisted of 1441 SIPs and 15,938 non-SIPs. In addition, we also created the yeast dataset employing the same strategy, which contained 710 positive sample SIPs and 5511 negative sample non-SIPs.

### Position specific scoring matrix

As a useful tool, position specific scoring matrix (PSSM) is formed through a set of sequences with structural or sequence similarity and was proposed by Gribskov et al. [25]. Meanwhile, it contains both the position information and evolutionary information of protein sequences, which is commonly employed to detect distantly related proteins. In addition, the PSSM is also widely applied in other areas such as prediction of membrane protein types [26], DNA-binding proteins prediction [27], prediction of protein structural classes [28], and drug-target interactions prediction [29] as well as obtaining excellent prediction results. Thus, considering that PSSM can preserve the evolutionary information of protein sequences as much as possible, we used it for predicting SIPs in this study. In this experiment, we employed Position Specific Iterated BLAST (PSI-BLAST) tool [30] to transform each protein sequence into a PSSM, and the vectors represented by these matrices can then be used to substitute protein sequences. Given a protein sequence, its PSSM may be represented as an $H \times 20$ matrix, which can be denoted as below:

1 $$M = \{ M_{\alpha \beta } :\alpha = 1 \ldots H,\beta = 1 \ldots 20\}$$

where the row H on the M matrix indicates the length of a given protein sequence, and 20 indicates a total of 20 amino acids due to the fact that each protein sequence consists of 20 types of amino acids. Next, for the query protein sequence, PSSM assigns a score $M_{\alpha \beta }$ to the $$\beta \begin{array}{*{20}c} {th} \\ \end{array}$$ amino acid in the $$\alpha \begin{array}{*{20}c} {th} \\ \end{array}$$ position by computing a position frequency matrix of each nucleotide in each position, so that the score $M_{\alpha \beta }$ can be represented as:

2 $$M_{\alpha \beta } = \sum\limits_{k = 1}^{20} {p(\alpha ,k)} \times q(\beta ,k)$$

where $p(\alpha ,k)$ stands for a matrix whose elements are the mutation value between two different amino acids, and $q(\beta ,k)$ stands for the value of the Dayhoff’s mutation matrix between $$\beta \begin{array}{*{20}c} {th} \\ \end{array}$$ and $$k\begin{array}{*{20}c} {th} \\ \end{array}$$ amino acids.

In summary, to create experimental datasets for predicting SIPs and obtain highly and widely homologous information, PSI-BLAST was employed to generate the PSSM for each protein sequence in this paper. Here, the e-value parameter and iteration number of PSI-BLAST were set to 0.001 and 3, respectively. Eventually, we can express the PSSM of each protein sequence as a 20-dimensional matrix consisting of $M \times 20$ elements.

### Adaptive synthetic sampling method

Adaptive synthetic (ADASYN), an oversampling approach of processing the minority classes, was first introduced by He et al. [31] for learning from imbalanced data sets. The essential idea of ADASYN is to employ a systematic approach, weighted distribution for specific minority categories of observations, to adaptively generate different numbers of synthetic observations in accordance with their distribution. By balancing original data sets with large differences between positive and negative samples, this algorithm can synthesize more observations from the minority classes that are relatively difficult to classify and fewer observations from the minority classes that are fairly easy to classify, thus addressing the class imbalance problem [32]. Not only does the ADASYN algorithm reduce the bias caused by class imbalance, but also adaptively shifts the classification decision boundary for the classifier toward those minority observations that are relatively difficult to classify.

Suppose $D_{o}$ is an original dataset consisting of *N* samples, in which $n_{s}$ denotes the number of observations in the minor class (interacting pairs) and $n_{l}$ denotes the number of observations in the major class (non-interacting pairs). The steps of ADASYN algorithm are briefly introduced below.

(1) Evaluate the level of class imbalance between samples below:

3 $$\begin{array}{*{20}c} {{{I = n_{s} } \mathord{\left/ {\vphantom {{I = n_{s} } {n_{l} }}} \right. \kern-\nulldelimiterspace} {n_{l} }},} & {} & {I \in } \\ \end{array} (0,1]$$

(2) To obtain new data $D_{n}$ for the whole minority class, the ADASYN approach will generate some minority class observations into the original data set $D_{o}$. The total number of synthetic observations to be generated can be computed as:

4 $$C = \alpha \left( {n_{l} - n_{s} } \right)$$

where $\alpha \in [0,1]$ is a parameter, which is employed to identify the desired balance level after generating the synthetic observations. If $\alpha { = }1,$ it means that a new data set whose samples of classes are fully balanced will be created after generating the synthetic observations.

(3) For every small instance $x_{i} ,$ $(x_{i} \in n_{s} )$ in each minority class, find its k-nearest neighbors in the n-dimensional space using the Euclidean distance method, and then we can calculate the ratio $r_{i}$ defined as:

5 $$r_{i} = {\Delta }_{i} /k,\quad i = 1, \ldots ,n_{s}$$

where $r_{i} \in [0,1]$ and ${\Delta }_{i}$ is the number of observations that are contained in the majority class, namely the k-nearest neighbors of $x_{i} .$

(4) Normalize the value of $r_{i}$ to a density distribution $\widehat{{r_{i} }}$ according to the following equation, while making the sum of all $\widehat{{r_{i} }}$ values equals 1.

6 $$\widehat{{r_{i} }} = r_{i} /\mathop \sum \limits_{i = 1}^{{n_{s} }} r_{i} \begin{array}{*{20}c} {} & {} & {\mathop \sum \limits_{i} \hat{r}_{i} = 1} \\ \end{array}$$

(5) For every small instance $x_{i}$ in the minor class, the number of synthetic observations which need to be generated is calculated as:

7 $$c_{i} = C\hat{r}_{i}$$

(6) By selecting an instance $x_{j}$ from the k-nearest neighbors of $x_{i}$ in the minor class, we finally can generate a new synthetic observation according to the following formula:

8 $$s_{i} = x_{i} + \left( {x_{j} - x_{i} } \right) \times \lambda$$

where $\lambda$ denotes a random number between 0 and 1, $x_{j}$ is one of the nearest neighbor observations of $x_{i} ,$ $\left( {x_{j} - x_{i} } \right)$ represents the difference vector in n-dimensional spaces, and $s_{i}$ is the new synthetic observation. Therefore, the ADASYN approach can automatically vote on the number of synthetic observations which need to be generated for each minority observation by utilizing a density distribution as a criterion.

### Gray level co-occurrence matrix (GLCM) features descriptor

In the process of classifying interacting and non-interacting protein pairs using a computational approach, a good feature extraction algorithm is essential for predicting SIPs efficiently and accurately. Although PSSM can effectively represent the evolutionary information of proteins, different proteins may contain different sequence lengths, which makes the size of the constructed PSSM inconsistent and cannot be directly used to compose the feature vectors of protein sequences. Thus, in this paper, we use GLCM to extract protein evolutionary information from PSSM to obtain the same length of feature vector descriptors. The GLCM algorithm, a classical texture-based feature extraction method, was introduced by Haralick et al. [33], which is widely employed in a variety of different tasks, especially for extracting spatial variation of the matrix in image processing applications. A GLCM is generated by computing the pixel brightness values (gray levels) that have specific values and a specified spatial relationship in an image. This spatial relationship is defined by a parameter pair $(\vartheta ,d)$ in which $\vartheta$ and $d$ represent the direction of two pixels and the separation distance between two pixels, respectively, which denotes the pixel of interest and the pixel that is horizontally adjacent to it. Typically, we need to define a set of parameter pairs $(\vartheta ,d)$ and combine them with GLCM matrices to describe the rotational invariance of the GLCM by employing a set of rotational parameters. Generally, this parameter is set to eight orientations and spaced to $\pi /4$ radians apart. The number of gray values $N_{g} ,$ as an integer, denotes the number of unique brightness values presented in the image. Normally, the image is scaled from $[0,255]$ to $[0,N_{g} ]$ before calculating the GLCM, where $N_{g}$ indicates the gray level and also determines the size of the gray level co-occurrence matrix [34].

In this experiment, the prominently used texture features of PSSM are extracted by using the GLCM algorithm, including contrast, correlation, energy, and homogeneity, which were defined by Haralick et al. [33]. The feature expressions of GLCM are shown below. Here, the variable $P(i,j)$ in each expression denotes the value at the $(i,j)th$ position in a grey level co-occurrence matrix.

9 $$contrast = \mathop \sum \nolimits_{i,j = 0}^{{N_{g} - 1}} (i - j)^{2} P(i,j)$$

10 $$correlation = \mathop \sum \nolimits_{i,j = 0}^{{N_{g} - 1}} \frac{{\left( {i - \mu_{x} } \right)\left( {j - \mu_{y} } \right)P(i,j)}}{{\sigma_{x} \sigma_{y} }}$$

where $\mu_{x} ,$ $\mu_{y} ,$ $\sigma_{x} ,$ $\sigma_{y}$ are the averages and the variances of the row and column, respectively, which are defined as follows:

11 $$\begin{array}{*{20}c} {\mu_{x} = \mathop \sum \nolimits_{i,j = 0}^{{N_{g} - 1}} i \cdot P(i,j)} & {} & {\mu_{y} = \mathop \sum \nolimits_{i,j = 0}^{{N_{g} - 1}} j \cdot P(i,j)} \\ \end{array}$$

12 $$\begin{array}{*{20}c} {\sigma_{x} = \sqrt {\mathop \sum \nolimits_{i,j = 0}^{{N_{g} - 1}} \left( {i - \mu_{x} } \right)^{2} \cdot P(i,j)} } & {} & {\sigma_{y} = \sqrt {\mathop \sum \nolimits_{i,j = 0}^{{N_{g} - 1}} \left( {j - \mu_{y} } \right)^{2} \cdot P(i,j)} } \\ \end{array}$$

13 $$energy = \mathop \sum \nolimits_{i,j = 0}^{{N_{g} - 1}} P(i,j)^{2}$$

14 $$homogeneity = \mathop \sum \nolimits_{i,j = 0}^{{N_{g} - 1}} \frac{P(i,j)}{{1 + (i - j)^{2} }}$$

As a result, we obtained a set of 60-dimensional statistical feature vectors from the PSSM of each protein sequence, using the GLCM feature extraction algorithm. In order to reduce the redundancy of features and computational burden, we first normalize all input feature vectors using the zero-mean normalization method. Second, considering the existence of unbalanced data samples, these feature vectors are fed into the ADASYN algorithm to generate new training samples to construct a relatively balanced data set so as to obtain an efficient and robust prediction model. Finally, the obtained new training features from the ADASYN algorithm are fed into the classification model for further feature classification.

### Weighted sparse representation based classification

Currently, machine learning algorithms used to construct classification models including Naive Bayes, decision trees and sparse representation classifiers, have been widely used in many fields. Sparse representation classifier (SRC), a popular nonparametric algorithm that is extensively applied in machine learning, was originally proposed by Wright et al. [35], which is analogous to the nearest neighbor and the nearest subspace approaches [36, 37]. The basic idea of SRC is to represent the individual test set by linearly combining the whole training set from original sample data. Then the sparsest representation of the individual test set is found in the dictionary. Finally, the new tests are assigned to the category with the minimum residual according to those representations. Although SRC has achieved good experimental results in many applications such as face recognition and text classification [38, 39], its prediction accuracy and classification effectiveness can be further enhanced. The SRC algorithm is briefly described as follows.

Considering a training instance set $T \in R^{d \times n} ,$ where $d$ means the dimension of feature vectors and $n$ means the number of training instances. Let $k$ denote the class number in the instance dataset. The $n_{k}$ instances belonging to the *kth* class can be expressed as a submatrix $T_{k} = \left[ {l_{k1} ,l_{k2} \ldots l_{{kn_{k} }} } \right],$ then the whole training set can be further rewritten as $$T = \left[ {\begin{array}{*{20}c} {T_{1} } & {T_{2} } & \ldots & {T_{K} } \\ \end{array} } \right],$$ where $K$ denotes the class number of the whole instance. Assuming that there is a new testing instance $x \in R^{d}$ belonging to the *kth* class, the sparse representation is to find such a column vector $\alpha = \left[ {\alpha_{k,1} ,\alpha_{k,2} , \ldots ,\alpha_{{k,n_{k} }} } \right]$ which satisfies the following condition:

15 $$x = \alpha_{k,1} l_{k,1} + \alpha_{k,2} l_{k,2} + \cdots + \alpha_{{k,n_{k} }} l_{{k,n_{k} }}$$

When representing the entire training instance set, this equation can be further rewritten as follows:

16 $$x = T\alpha_{0}$$

According to the sparse representation method, we note that the nonzero entries in $\alpha_{0}$ are only related to the *kth* class, which can be denoted as

17 $$\alpha_{0} = \left[ {0, \cdots ,0,\alpha_{k,1} ,\alpha_{k,2} \cdots \alpha_{{k,n_{k} }} ,0, \cdots ,0} \right]^{T}$$

Next, in the SRC algorithm, we need to solve the following $l_{0}$-norm minimization problem:

18 $$\begin{array}{*{20}c} {\hat{\alpha }_{0} = \arg \min \parallel \alpha \parallel_{0} } & {} & {{\text{subject}}} \\ \end{array} {\text{ to }}x = T\alpha$$

Since the solution of $\hat{\alpha }_{0}$ is an NP-hard problem, we need to optimize this problem. When $\alpha$ is sufficiently sparse, the problem can be solved in this way by solving the $l_{1}$-minimization problem instead of solving the $l_{0}$-minimization directly.

19 $$\begin{array}{*{20}c} {\hat{\alpha }_{1} = \arg \min \parallel \alpha \parallel_{1} } & {} & {{\text{subject}}} \\ \end{array} {\text{ to }}x = T\alpha$$

To avoid the occlusion problem and enhance the generalization capability of the SRC algorithm, the $l_{1}$-norm minimization is further extended to the following stable $l_{1}$-norm minimization problem by introducing $\varepsilon ,$ a threshold of the reconstruction error.

20 $$\begin{array}{*{20}c} {\hat{\alpha }_{1} = \arg \min \parallel \alpha \parallel_{1} } & {} & {{\text{subject}}} \\ \end{array} {\text{ to }}\parallel x - T\alpha \parallel \le \varepsilon$$

Subsequently, the given test instance $x$ is assigned to class $k$ by computing the smallest reconstruction residual, which can be expressed as follows:

21 $$r_{k} (x) = \parallel x - T\hat{\alpha }_{1}^{k} \parallel ,\quad k = 1,2, \ldots ,K$$

where $T\hat{\alpha }_{1}^{k}$ is the reconstructed value that is obtained from the training instance of class $k,$ $r_{k}$ denotes the residual, and $K$ is the class number of the whole instance. Finally, the class with the minimum residual will be obtained, which will be used as the prediction label of the test instance $x.$

In this study, a new classification model, weighted sparse representation-based classifier (WSRC), is used to predict SIPs based on a novel feature extraction description of protein sequences. WSRC is a variant of the traditional sparse representation classifier, which can enhance the classification performance of prediction models [40]. WSRC utilizes the distance information to represent the test samples and assigns weights to the samples in the training set, whereas the typical SRC does not explore the distance or similarity relationship from individual training samples to the test samples. However, previous research has confirmed that the locality of data is also more essential than sparsity in some cases [41, 42]. According to this assumption, the WSRC model needs to integrate the locality structure of the data based on the traditional sparse representation in order to evaluate the importance of each training instance in representing the testing instance. WSRC employs the Gaussian kernel distance that can capture the nonlinear information within the original dataset to calculate the weights. For the given two instances, $s_{1}$ and $s_{2} ,$ the distance based on the Gaussian kernel between them is as follows:

22 $$d_{G} \left( {s_{1} ,s_{2} } \right) = e^{{ - \parallel s_{1} - s_{2} \parallel^{2} /2\sigma^{2} }}$$

where $\sigma$ is the Gaussian kernel width that needs to be specified in advance in the experiment. Using Gaussian kernel distance as a nonlinear mapping to compute weights, WSRC can effectively capture the locally nonlinear information within the dataset. By this way, the WSRC algorithm will turn to solve the following $l_{1}$-norm minimization problem:

23 $$\begin{array}{*{20}c} {\hat{\alpha }_{1} = \arg \min \parallel W\alpha \parallel_{1} } & {} & {{\text{subject}}} \\ \end{array} {\text{ to }}x = T\alpha$$

and more specifically,

24 $$diag(W) = \left[ {d_{G} \left( {x,t_{1}^{1} } \right), \ldots ,d_{G} \left( {x,t_{{n_{k} }}^{k} } \right)} \right]^{T}$$

where $W$ is a block-diagonal matrix and $n_{k}$ is the number of the training samples in the *kth* class. Similarly, to simplify this problem, the WSRC algorithm will further turn to solve the stable $l_{1}$-norm minimization problem, which can be expressed as follows:

25 $$\begin{array}{*{20}c} {\hat{\alpha }_{1} = \arg \min \parallel W\alpha \parallel_{1} } & {} & {{\text{subject}}} \\ \end{array} {\text{ to }}\parallel x - T\alpha \parallel \le \varepsilon$$

where $\varepsilon > 0$ is a threshold, namely the tolerance value of the reconstruction error using a linear combination of the training samples to denote the test samples.

## Results and discussion

### Performance evaluation

In order to assess the effectiveness and feasibility of the proposed method in this paper, we used the following measures, namely accuracy (Acc.), sensitivity (Sen.), specificity (Spe.), precision (Pre.) and Matthews correlation coefficient (MCC), as the prediction performance indicators of the model in this experiment, which are expressed as:

26 $$Acc. = \frac{TP + TN}{{TP + TN + FP + FN}}$$

27 $$Sen. = \frac{TP}{{FN + TP}}$$

28 $$Spe. = \frac{TN}{{FP + TN}}$$

29 $$Pre. = \frac{TP}{{TP + FP}}$$

30 $$MCC = \frac{TP \times TN - FP \times FN}{{\sqrt {{(}TP + FP{)} \times {(}TP + FN{)} \times {(}TN + FP{)} \times {(}TN + FN{)}} }}$$

**Fig. 1:**
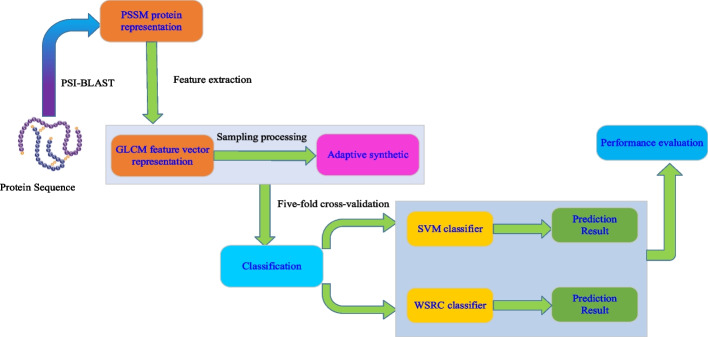
Flow chart of the proposed model for predicting potential SIPs

In the above formula, where *TP* represents true positives, meaning the count of those samples that have interacted pairs are predicted correctly by the model, *FP* represents false positives, meaning the number of those samples that are true non-interacting pairs are judged to be interacting pairs by the model, *TN* represents true negatives, meaning the number of those samples that have true non-interacting pairs are predicted correctly by the model, and *FN* represents false negatives, meaning the count of those samples that are true interacting pairs are judged to be non-interacting pairs by the model. Additionally, to clearly visualize the performance of our model for predicting SIPs classification results, we also plotted the receiver operating characteristic curve (ROC) and computed the AUC (area under the ROC) and the AUPR (area under the precision-recall curve) as an important evaluation metric [43]. The main workflow of the proposed model is shown in Fig. 1.

### Performance of the proposed method

In this study, we used two standard datasets, namely yeast and human for validating the performance of the proposed model in predicting SIPs. For the sake of preventing the overfitting phenomenon affecting the prediction results as much as possible, we employed a five-fold cross-validation method in the experiment and separated the original SIPs experimental dataset into the training set and independent test set. Here, taking the yeast dataset as an example, we described it in detail by splitting the entire dataset into five non-overlapping parts, where 4 parts are used as training samples and the remaining 1 part is taken as testing samples. After this, we can obtain five separate models and can then perform five separate SIPs experiments utilizing the proposed method. In the end, the experimental results achieved by our method on the yeast dataset were represented as the mean and standard deviation of five predicted outcomes. Similarly, the same strategy was also applied to the human dataset.

**Fig. 2:**
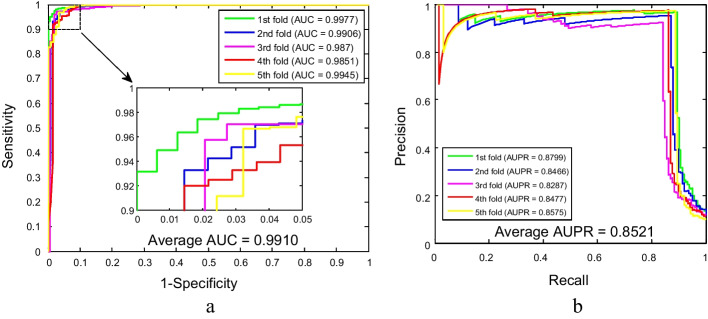
The ROC and AUPR performance of WSRC-based method on yeast SIPs dataset

**Fig. 3:**
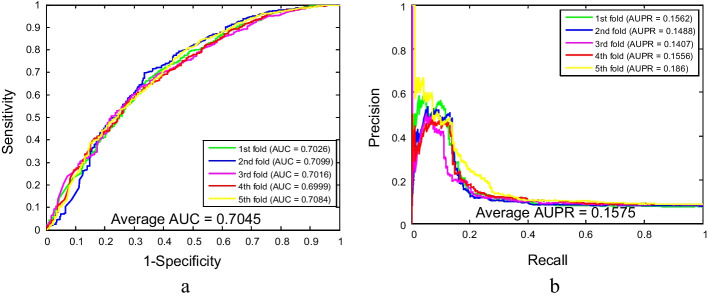
The ROC and AUPR performance of WSRC-based method on human SIPs dataset

As shown in Tables 1 and 2, the proposed method for predicting SIPs combined with five-fold cross-validation yielded satisfying results on yeast and human datasets. From Table 1, it is evident that the overall accuracies of these five experiments are above 97% for the yeast dataset. More specifically, the accuracies of five experiments are 98.23%, 98.07%, 97.35%, 98.23% and 98.63%, respectively. The average accuracy is 98.10%, the average sensitivity is 87.17%, the average specificity is 99.51%, the average precision is 95.87%, and the average MCC is 90.51%, with standard deviations of 0.47%, 2.08%, 0.26%, 2.09% and 2.09%, respectively. Similarly, our method also obtained better experimental results on larger human datasets. The accuracies of each experiment are 92.40%, 91.80%, 91.19%, 91.02% and 91.15%, respectively. The values of average accuracy, sensitivity, specificity, precision and MCC are 91.51%, 12.75%, 98.63%, 46.11% and 25.31%, respectively, with the corresponding standard deviations of 0.58%, 1.40%, 0.28%, 4.14% and 1.58%, respectively. Meanwhile, the ROC and AUPR curves plotted by our model for detecting SIPs on yeast and human datasets are shown in Figs. 2 and 3. Among them, the average AUC values of the model on yeast and human datasets are 99.10% and 70.45%, respectively, and the average AUPR values of the model on yeast and human datasets are 85.21% and 15.75%, respectively. In these figures, y-axis and x-axis refer to true positive rate (TPR) and false positive rate (FPR), respectively. In addition, these high assessment standard values and relatively small standard deviations in the experimental results indicate that the proposed method, as a computational method, is accurate and reliable in predicting SIPs.

**Table 1:** Five-fold cross-validation prediction results using the WSRC-based method on the yeast dataset

Testing set	Acc. (%)	Sen. (%)	Spe. (%)	Pre. (%)	MCC (%)
1	98.23	89.51	99.54	96.67	92.13
2	98.07	87.14	99.46	95.31	90.22
3	97.35	84.25	99.09	92.48	87.10
4	98.23	86.23	99.73	97.54	90.85
5	98.63	88.71	99.73	97.35	92.26
Average	98.10 ± 0.47	87.17 ± 2.08	99.51 ± 0.26	95.87 ± 2.09	90.51 ± 2.09

**Table 2:** Five-fold cross-validation prediction results using the WSRC-based method on the human dataset

Testing set	Acc. (%)	Sen. (%)	Spe. (%)	Pre. (%)	MCC (%)
1	92.40	11.61	99.13	52.54	25.32
2	91.80	14.03	98.56	45.88	26.46
3	91.19	10.96	98.55	41.03	22.64
4	91.02	13.20	98.46	44.94	25.61
5	91.15	13.95	98.46	46.15	26.54
Average	91.51 ± 0.58	12.75 ± 1.40	98.63 ± 0.28	46.11 ± 4.14	25.31 ± 1.58

### Prediction performance of the support vector machine-based method

It is remarkable that the proposed model achieved better prediction performance on two benchmark datasets and can be used to detect SIPs fairly well. However, Support Vector Machine (SVM), as a widespread data mining algorithm, has strong practicality both in machine learning and in pattern recognition, which has an excellent prediction performance especially in dealing with classification and regression problems [44]. Hence, to better understand the predictive performance of our classifier, we try to adopt the most popular SVM instead of WSRC to perform SIPs, which is a comparison experiment for the proposed method. Specifically, the same feature extraction method was employed in conjunction with the SVM classifier to execute the prediction of SIPs on yeast and human datasets, respectively. Here, we carry out the SVM classification task employing the LIBSVM toolbox [45], which can be downloaded from website https://www.csie.ntu.edu.tw/~cjlin/libsvm/. For ensuring fairness, we optimized the relevant parameters of the SVM by selecting the radial basis function as the kernel function. The parameters c and g were set to 0.5 and 1, respectively, on the yeast dataset, and the parameters c and g were set to 4 and 8, respectively, on the human dataset, which was determined by using the grid search method and other parameters were set as default values.

**Fig. 4:**
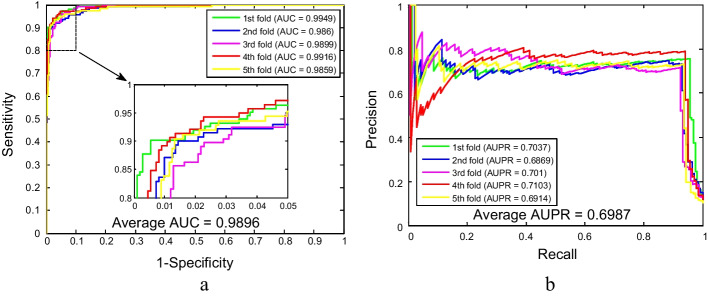
The ROC and AUPR performance of SVM-based method on yeast SIPs dataset

**Fig. 5:**
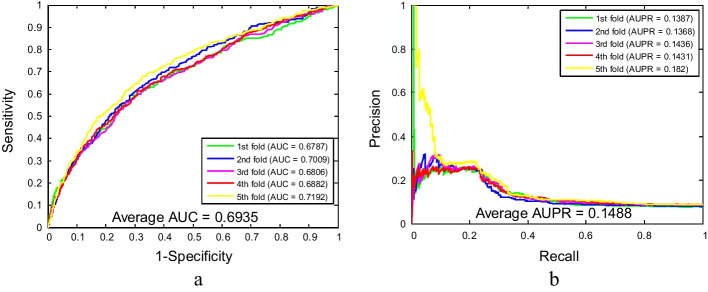
The ROC and AUPR performance of SVM-based method on human SIPs dataset

Tables 3 and 4 summarized the predicted results of SIPs using two classifiers in combination with the fivefold cross-validation method on the yeast and human datasets. As seen from Table 3, the GLCM-SVM method obtained an average accuracy of 95.60% on the yeast dataset, wherein the accuracies of the five models are 95.42%, 95.34%, 94.77%, 96.54%, and 95.90% respectively. Nevertheless, the GLCM-WSRC approach achieved an average accuracy of 98.10% in predicting SIPs, which is indeed 2.5% larger than the average accuracy gained by the SVM approach. Likewise, as shown in Table 4, the GLCM-SVM method yielded an average accuracy of 87.86% on the human dataset, of which the accuracies of the five experiments are 88.40%, 87.74%, 87.71%, 87.42%, and 88.01% respectively. The highest accuracy rate obtained based on the SVM model is 2.62% lower than the minimum accuracy rate achieved based on the WSRC model as compared to the predicted results of the WSRC method on the human dataset. The predictive performance of the ROC and AUPR curves based on the SVM method on the yeast and human datasets is shown in Figs. 4 and 5. Among them, the average AUC values of the model on yeast and human datasets are 98.96% and 69.35%, respectively, and the average AUPR values of the model on yeast and human datasets are 69.87% and 14.88%, respectively. Consequently, it can be seen from these evaluation metrics that the experimental results obtained by the WSRC classifier are superior to those obtained by the SVM classifier in detecting SIPs, which further indicates that the proposed computational model can provide a useful supplementary tool for predicting SIPs, as well as other bioinformatics tasks.

**Table 3:** Five-fold cross-validation prediction results using the SVM-based method on the yeast dataset

Model	Testing set	Acc. (%)	Sen. (%)	Spe. (%)	Pre. (%)	MCC (%)
GLCM-SVM	1	95.42	95.68	95.38	75.61	82.97
	2	95.34	92.14	95.74	73.30	80.30
	3	94.77	92.47	95.08	71.43	79.16
	4	96.54	94.20	96.84	78.79	84.63
	5	95.90	93.55	96.16	72.96	80.94
	Average	95.60 ± 0.66	93.61 ± 1.42	95.84 ± 0.69	74.42 ± 2.87	81.60 ± 2.19
GLCM-WSRC	Average	98.10 ± 0.47	87.17 ± 2.08	99.51 ± 0.26	95.87 ± 2.09	90.51 ± 2.09

**Table 4:** Five-fold cross-validation prediction results using the SVM-based method on the human dataset

Model	Testing set	Acc. (%)	Sen. (%)	Spe. (%)	Pre. (%)	MCC (%)
GLCM-SVM	1	88.40	23.60	93.80	24.05	27.11
	2	87.74	24.46	93.24	23.94	27.64
	3	87.71	22.95	93.65	24.91	27.38
	4	87.42	22.77	93.60	25.37	27.58
	5	88.01	22.59	94.21	26.98	27.94
	Average	87.86 ± 0.37	23.27 ± 0.76	93.70 ± 0.35	25.05 ± 1.23	27.53 ± 0.31
GLCM-WSRC	Average	91.51 ± 0.58	12.75 ± 1.40	98.63 ± 0.28	46.11 ± 4.14	25.31 ± 1.58

### Comparison with other methods

Currently, several computational models based on protein sequences have been proposed to detect SIPs. In this section, to further objectively evaluate the superior performance of our model, we compared it with the following six methods that have been shown to achieve a good prediction performance on the same two standard datasets. These existing methods include SLIPPER [46], CRS [18], SPAR [18], DXECPPI [47], PPIevo [12] and LocFuse [48], which are all classical methods designed to detect SIPs. The comparison results between the proposed method and these six methods are clearly given in Tables 5 and 6, which indicated the good performance of our method, using ADASYN algorithm, GLCM feature extraction, and WSRC classifier to predict SIPs in this paper, as compared to the previous computational methods. From Table 5, we can see that the proposed method yielded a high accuracy of 98.10% when detecting the SIPs of the yeast dataset, which is significantly higher than the six existing methods obtaining relatively low accuracies between 66.28% and 87.46%. Furthermore, compared with the other methods, the proposed method achieves relatively low standard deviations with respect to these evaluation metrics, which also implies that our model has a more robust predictive performance in predicting SIPs. At the same instant, we obtained relatively good prediction results from Table 6 when detecting the SIPs of the human dataset. The accuracy of the proposed predictor is 91.51%, which is 60.61% higher than the DXECPPI method, 13.47% higher than the PPIevo method, and 10.85% higher than the LocFuse method. These comparison results further demonstrate that the proposed method is capable of efficiently predicting SIPs from protein sequences.

**Table 5:** Predictive performance of seven different methods on yeast dataset

Model	Acc. (%)	Spe. (%)	Sen. (%)	MCC (%)
SLIPPER	71.90	72.18	69.72	28.42
DXECPPI	87.46	94.93	29.44	28.25
PPIevo	66.28	87.46	60.14	18.01
LocFuse	66.66	68.10	55.49	15.77
CRS	72.69	74.37	59.58	23.68
SPAR	76.96	80.02	53.24	24.84
Proposed method	98.10	99.51	87.17	90.51

**Table 6:** Predictive performance of seven different methods on human dataset

Model	Acc. (%)	Spe. (%)	Sen. (%)	MCC (%)
SLIPPER	91.10	95.06	47.26	41.97
DXECPPI	30.90	25.83	87.08	8.25
PPIevo	78.04	25.82	87.83	20.82
LocFuse	80.66	80.50	50.83	20.26
CRS	91.54	96.72	34.17	36.33
SPAR	92.09	97.40	33.33	38.36
Proposed method	91.51	98.63	12.75	25.31

The main reasons for the proposed method yielding better prediction results come from the following points: (1) PSSM can contain protein evolutionary information. (2) The GLCM feature extraction algorithm can accurately capture useful knowledge from the PSSM. (3) The ADASYN method can efficiently integrate training features to balance the training sample set and reduce the impact of noise. (4) WSRC can effectively discover differences between different types and improve the prediction performance for the classification tasks dealing with interacting and non-interacting proteins, by integrating both sparsity and data locality structure into traditional SRC. (5) Compared with other methods, WSRC can automatically obtain a good prediction result in detecting SIPs, which is mainly due to the fact that it could keep the same parameters in all SIPs experiments and does not require much manual intervention to adjust its parameters.

## Conclusion

Recently, the rise of machine learning techniques constantly promotes highly interdisciplinary research in different fields such as protein–protein interaction prediction, drug-target interaction prediction, and drug-disease association prediction. In this study, we present a novel computational method named GLCM-WSRC, which combines GLCM feature extraction algorithm, ADASYN technique with WSRC classification model for predicting SIPs based on protein evolutionary information from protein primary sequences. Specifically, each protein sequence was transformed into a PSSM, a two-dimensional matrix that can contain protein evolutionary information, by using the PSI-BLAST method. The GLCM algorithm is then employed to capture the valuable information from these PSSMs and form feature vectors of the proteins, after which the ADASYN technique is applied to balance the training data set to form new feature vectors used as the input of classifier from the obtained GLCM feature vectors. Finally, the weighted sparse representation based classification machine learning strategy is adopted to predict SIPs. Promising experimental results reveal that the constructed model is feasible and efficient when dealing with the classification task of interacting and non-interacting pairs of protein sequences, which achieves better prediction performances than other existing works on the same benchmark dataset. Thus, our work provides a useful tool for large-scale self-interacting protein prediction, which is beneficial for the detection of other bioinformatics tasks in the future.

## Data Availability

The datasets used and/or analyzed during the current study are available from the author on reasonable requests.

## Abbreviations

SIPs: Self-interacting proteins
GLCM-WSRC: Gray level co-occurrence matrix-weighted sparse representation based classification
PSSM: Position Specific Scoring Matrix
PSI-BLAST: Position Specific Iterated BLAST
ADASYN: Adaptive synthetic
PPIs: Protein–protein interactions
PINs: Protein interaction networks
SVM: Support vector machine
Bio2Vec: Bio-to-vector
CNN: Convolution neural network
SPAR: Self-interacting Protein Analysis serveR
CRS: Critical residues substitution
GLCM: Gray level co-occurrence matrix
WSRC: Weighted sparse representation based classification
Acc.: Accuracy
Sen.: Sensitivity
Spe.: Specificity
Pre.: Precision
MCC: Matthews correlation coefficient
ROC: Receiver operating characteristic curve
AUC: Area under the ROC
AUPR: Area under the precision-recall curve
TPR: True positive rate
FPR: False positive rate
SRC: Sparse representation classifier

## References

[1] Chen Y, Dokholyan NV (2008). Natural selection against protein aggregation on self-interacting and essential proteins in yeast, fly, and worm. Mol Biol Evol.

[2] Li Y, Wang Z, Li L-P, You Z-H, Huang W-Z, Zhan X-K, Wang Y-B (2021). Robust and accurate prediction of protein–protein interactions by exploiting evolutionary information. Sci Rep.

[3] Koike R, Kidera A, Ota M (2009). Alteration of oligomeric state and domain architecture is essential for functional transformation between transferase and hydrolase with the same scaffold. Protein Sci.

[4] Baisamy L, Jurisch N, Diviani D (2005). Leucine zipper-mediated homo-oligomerization regulates the Rho-GEF activity of AKAP-Lbc. J Biol Chem.

[5] Katsamba P, Carroll K, Ahlsen G, Bahna F, Vendome J, Posy S, Rajebhosale M, Price S, Jessell T, Ben-Shaul A (2009). Linking molecular affinity and cellular specificity in cadherin-mediated adhesion. Proc Natl Acad Sci.

[6] An J-Y, Zhou Y, Yan Z-J, Zhao Y-J (2020). Predicting self-interacting proteins using a recurrent neural network and protein evolutionary information. Evol Bioinforma.

[7] Li J-Q, You Z-H, Li X, Ming Z, Chen X (2017). PSPEL: in silico prediction of self-interacting proteins from amino acids sequences using ensemble learning. IEEE/ACM Trans Comput Biol Bioinf.

[8] Chen Z-H, You Z-H, Li L-P, Wang Y-B, Wong L, Yi H-C (2019). Prediction of self-interacting proteins from protein sequence information based on random projection model and fast Fourier transform. Int J Mol Sci.

[9] Chen C, Zhang Q, Yu B, Yu Z, Lawrence PJ, Ma Q, Zhang Y (2020). Improving protein–protein interactions prediction accuracy using XGBoost feature selection and stacked ensemble classifier. Comput Biol Med.

[10] Wang Y, You Z, Li L, Chen Z (2020). A survey of current trends in computational predictions of protein–protein interactions. Front Comp Sci.

[11] Wang Y, You Z, Li X, Chen X, Jiang T, Zhang J (2017). PCVMZM: using the probabilistic classification vector machines model combined with a zernike moments descriptor to predict protein–protein interactions from protein sequences. Int J Mol Sci.

[12] Zahiri J, Yaghoubi O, Mohammad-Noori M, Ebrahimpour R, Masoudi-Nejad A (2013). PPIevo: protein–protein interaction prediction from PSSM based evolutionary information. Genomics.

[13] Huang Y-A, You Z-H, Chen X, Chan K, Luo X (2016). Sequence-based prediction of protein-protein interactions using weighted sparse representation model combined with global encoding. BMC Bioinformatics.

[14] Xia J-F, Han K, Huang D-S (2010). Sequence-based prediction of protein-protein interactions by means of rotation forest and autocorrelation descriptor. Protein Pept Lett.

[15] An J-Y, You Z-H, Zhou Y, Wang D-F (2019). Sequence-based prediction of protein-protein interactions using gray wolf optimizer–based relevance vector machine. Evol Bioinforma.

[16] Shi M-G, Xia J-F, Li X-L, Huang D-S (2010). Predicting protein–protein interactions from sequence using correlation coefficient and high-quality interaction dataset. Amino Acids.

[17] Wang Y, You Z-H, Yang S, Li X, Jiang T-H, Zhou X (2019). A high efficient biological language model for predicting protein–protein interactions. Cells.

[18] Liu X, Yang S, Li C, Zhang Z, Song J (2016). SPAR: a random forest-based predictor for self-interacting proteins with fine-grained domain information. Amino Acids.

[19] Consortium U (2015). UniProt: a hub for protein information. Nucleic Acids Res.

[20] Chatr-Aryamontri A, Breitkreutz B-J, Oughtred R, Boucher L, Heinicke S, Chen D, Stark C, Breitkreutz A, Kolas N, O'Donnell L (2015). The BioGRID interaction database: 2015 update. Nucleic Acids Res.

[21] Salwinski L, Miller CS, Smith AJ, Pettit FK, Bowie JU, Eisenberg D (2004). The database of interacting proteins: 2004 update. Nucleic Acids Res.

[22] Breuer K, Foroushani AK, Laird MR, Chen C, Sribnaia A, Lo R, Winsor GL, Hancock RE, Brinkman FS, Lynn DJ (2013). InnateDB: systems biology of innate immunity and beyond—recent updates and continuing curation. Nucleic Acids Res.

[23] Orchard S, Ammari M, Aranda B, Breuza L, Briganti L, Broackes-Carter F, Campbell NH, Chavali G, Chen C, Del-Toro N (2014). The MIntAct project—IntAct as a common curation platform for 11 molecular interaction databases. Nucleic Acids Res.

[24] Clerc O, Deniaud M, Vallet SD, Naba A, Rivet A, Perez S, Thierry-Mieg N, Ricard-Blum S (2019). MatrixDB: integration of new data with a focus on glycosaminoglycan interactions. Nucleic Acids Res.

[25] Gribskov M, McLachlan AD, Eisenberg D (1987). Profile analysis: detection of distantly related proteins. Proc Natl Acad Sci.

[26] Hayat M, Khan A (2012). MemHyb: predicting membrane protein types by hybridizing SAAC and PSSM. J Theor Biol.

[27] Zhang S, Zhu F, Yu Q, Zhu X (2021). Identifying DNA-binding proteins based on multi-features and LASSO feature selection. Biopolymers.

[28] Liang Y, Liu S, Zhang S (2015). Prediction of protein structural classes for low-similarity sequences based on consensus sequence and segmented PSSM. Comput Math Methods Med.

[29] Wang L, You Z-H, Chen X, Yan X, Liu G, Zhang W (2018). Rfdt: a rotation forest-based predictor for predicting drug-target interactions using drug structure and protein sequence information. Curr Protein Pept Sci.

[30] Li Y, Liu XZ, You ZH, Li LP, Guo JX, Wang Z (2021). A computational approach for predicting drug–target interactions from protein sequence and drug substructure fingerprint information. Int J Intell Syst.

[31] He H, Bai Y, Garcia EA, Li S. ADASYN: adaptive synthetic sampling approach for imbalanced learning. In: 2008 IEEE international joint conference on neural networks (IEEE world congress on computational intelligence). IEEE, 2008, p. 1322–8.

[32] He H, Garcia EA (2009). Learning from imbalanced data. IEEE Trans Knowl Data Eng.

[33] Haralick RM, Shanmugam K, Dinstein IH (1973). Textural features for image classification. IEEE Trans Syst Man Cybern.

[34] Lohithashva B, Aradhya VM, Guru D (2020). Violent video event detection based on integrated LBP and GLCM texture features. Rev d'Intell Artif.

[35] Wright J, Yang AY, Ganesh A, Sastry SS, Ma Y (2008). Robust face recognition via sparse representation. IEEE Trans Pattern Anal Mach Intell.

[36] Lee K-C, Ho J, Kriegman DJ (2005). Acquiring linear subspaces for face recognition under variable lighting. IEEE Trans Pattern Anal Mach Intell.

[37] Li SZ. Face recognition based on nearest linear combinations. In: Proceedings of 1998 IEEE computer society conference on computer vision and pattern recognition (Cat. No. 98CB36231). IEEE. 1998; p. 839–44.

[38] Ye M-J, Hu C-H, Wan L-G, Lei G-H (2021). Fast single sample face recognition based on sparse representation classification. Multimed Tools Appl.

[39] Unnikrishnan P, Govindan V, Kumar SM (2019). Enhanced sparse representation classifier for text classification. Expert Syst Appl.

[40] Lu C-Y, Min H, Gui J, Zhu L, Lei Y-K (2013). Face recognition via weighted sparse representation. J Vis Commun Image Represent.

[41] Wang J, Yang J, Yu K, Lv F, Huang T, Gong Y. Locality-constrained linear coding for image classification. In: 2010 IEEE Computer Society Conference on Computer Vision and Pattern Recognition. IEEE. 2010; p. 3360–3367.

[42] Roweis ST, Saul LK (2000). Nonlinear dimensionality reduction by locally linear embedding. Science.

[43] Zhao B-W, You Z-H, Hu L, Guo Z-H, Wang L, Chen Z-H, Wong L (2021). A novel method to predict drug-target interactions based on large-scale graph representation learning. Cancers.

[44] Tahir M, Jan B, Hayat M, Shah SU, Amin M (2018). Efficient computational model for classification of protein localization images using extended threshold adjacency statistics and support vector machines. Comput Methods Programs Biomed.

[45] Chang C-C, Lin C-J (2011). LIBSVM: a library for support vector machines. ACM Trans Intell Syst Technol.

[46] Liu Z, Guo F, Zhang J, Wang J, Lu L, Li D, He F (2013). Proteome-wide prediction of self-interacting proteins based on multiple properties. Mol Cell Proteomics.

[47] Du X, Cheng J, Zheng T, Duan Z, Qian F (2014). A novel feature extraction scheme with ensemble coding for protein–protein interaction prediction. Int J Mol Sci.

[48] Zahiri J, Mohammad-Noori M, Ebrahimpour R, Saadat S, Bozorgmehr JH, Goldberg T, Masoudi-Nejad A (2014). LocFuse: human protein–protein interaction prediction via classifier fusion using protein localization information. Genomics.

